# Relationship between 24-h activity behavior and body fat percentage in preschool children: based on compositional data and isotemporal substitution analysis

**DOI:** 10.1186/s12889-024-18570-2

**Published:** 2024-04-16

**Authors:** Jinmei Fu, Shunli Sun, Shenggen Zhu, Runze Wang, Delong Chen, Ruiming Chen, Ran Xue, Wendi Lv, Yunfan Zhang, Ting Huang, Xuewen Hu, Tianle Jiang, Lei Wen, Liqiang Su, Zihao He, Guanggao Zhao, Weilu Zou

**Affiliations:** 1 Jiangxi Province Sports Science Medical Center, No.28 Fuzhou Road, Nanchang, 330006 Jiangxi China; 2 Physical Education Institute, Xinyu University, No.2666 Sunshine Avenue, Xinyu, 330038 Jiangxi China; 3 Physical Education Institute, Nanchang University, No.999 Xuefu Avenue, Nanchang, 330038 Jiangxi China; 4 School of Sports and Health, Jiangxi University of Traditional Chinese Medicine, 1688 Meiling Avenue, Nanchang, 330004 NoJiangxi China; 5 School of Physical Education and Sport Science, Science and Technology Road, Fujian Normal University, No.1, University Town, 350117 Fujian, China; 6 Physical Education Institute, Jiangxi Normal University, No.99 Ziyang Avenue, Nanchang, 330022 Jiangxi China; 7 College of Sports and Human Sciences, Beijing Sport University, No.48 Information Road, Beijing, 100091 China; 8 Jiangxi Provincial Gymnastics Sports Management Center, 28 Fuzhou Road, Nanchang, 330006 Jiangxi China

**Keywords:** Preschool children, 24 h activity, Body fat percentage, Component analysis, Isochronous substitution

## Abstract

**Objective:**

This study aims to elucidate the dose‒response relationship between 24-h activity behaviors and body fat percentage (BFP) in Chinese preschool children using a compositional isotemporal substitution model (ISM).

**Methods:**

In a cross-sectional design, 881 children aged 3–6 from urban and rural areas of Jiangxi Province were sampled. Activity behaviors, including sedentary behavior (SB), low-intensity physical activity (LPA), and moderate- to high-intensity physical activity (MVPA), were measured using accelerometers. Sleep patterns were assessed through questionnaires, and BFP was determined by bioelectrical impedance analysis (BIA). The study employed compositional data analysis (CoDA) and ISM to estimate the impact of reallocating durations of different activity behaviors on BFP.

**Results:**

Higher BFP was found in urban vs. rural children, decreasing with age. Overweight and obesity rates were 10.6% and 7.6%, respectively, above national averages. MVPA and LPA were negatively correlated with BFP, while SB was positively correlated.

A 30-min MVPA reduction significantly increased zBFR, particularly in overweight children. Gender-specific nuances revealed that boys' MVPA negatively influenced zBFP (*β* = -0.155), *P* < 0.05), while girls' SB positively impacted zBFP (*β* = 0.636, *P* < *0.01*). Isotemporal simulations emphasized amplified effects in overweight children, with boys' zBFR rising rapidly when MVPA was substituted and girls displaying a notable substitution effect between SB and LPA.

**Conclusion:**

BFP is closely linked to 24-h activity behaviors, notably in overweight and obese preschoolers. ISM identified MVPA as a critical influencer, with a 30-min reduction substantially increasing BFP. Gender disparities were evident, implicating MVPA in boys and LPA and SB in girls.

## Introduction

Childhood overweight and obesity are growing global health concerns, particularly among preschoolers. The World Health Organization reported that in 2020, over 39 million children under five were overweight or obese, making up 5.7% of this demographic [1]. Recent data indicates that in China, the prevalence among preschool children has risen sharply to 10.4% [2]. These trends pose risks to children's physical and psychological well-being and increase their vulnerability to obesity and associated chronic diseases later in life [3–5]. Identifying and addressing the determinants of overweight and obesity in this age group is crucial for effective prevention and management.

Given global health promotion strategies, it's essential to understand the relationship between physical activity (PA), sedentary behavior (SB), and sleep (SP) in the context of overweight and obesity. While adequate PA can significantly reduce the risk of these conditions [6–10], many preschool children in China are not sufficiently active [11]. Balancing PA, SB, and SP to maximize health benefits is a current challenge.

Considering daily activity duration remains constant, altering the duration of one activity will affect another. Instead of examining these behaviors separately, they should be viewed as an interconnected system, treating 24-hour activity data as compositional. Traditional studies treated SP, SB, LPA, and MVPA as independent variables [6–9], while more recent research recognized their interrelated effects [5, 12–14]. However, the latter often faced multicollinearity issues in regression analysis. Ignoring the compositional nature of 24-hour activity data can lead to inconsistent findings [15].

Compositional data analysis (CoDA) addresses these issues by treating 24-hour activity behavior as compositional data, allowing for transformations from simplex to Euclidean space [16–18]. CoDA can also highlight the combined effects of 24-hour activity behaviors on health outcomes [19, 20]. The isotemporal substitution model (ISM) further examines the health implications of substituting one activity for another [21]. By understanding the balance of 24-hour activity behavior, we can derive insights into the relationship between PA behaviors and health outcomes, leading to practical clinical guidelines.

While several international studies have investigated the influence of 24-hour activity behavior on childhood obesity and overweight [22–24], findings, particularly for preschool children, have been inconsistent [25, 26]. In China, most research in this area has relied on Body Mass Index (BMI) and its derivatives, such as zBMI scores [12]. Furthermore, the potential health effects resulting from substituting different types of activities vary across age groups. Preschool children exhibit distinct activity behavior patterns compared to older children and adolescents [27]. Therefore, understanding how to balance physical activity, sedentary behavior, and sleep to maximize the health benefits of exercise is a pressing issue that this study aims to address.

This study aims to use the compositional iso-temporal substitution model to explore the relationship between 24-hour activity behavior and body fat percentage in preschool children. This research is anticipated to contribute to the foundation for developing customized PA guidelines for preschool children. It aims to provide insights that could be instrumental in shaping approaches to tackle obesity trends among this age group. These insights are expected to inform the development of interventions, aligning with the broader goal of fostering healthier lifestyles in preschool children in China.

## Methods

### Study design and participants

This cross-sectional study received ethical approval from the Ethics Committee of the Second Affiliated Hospital of Nanchang University (Approval No. 125, 2020), and participation in the study was contingent upon obtaining informed consent from both the parents and the kindergartens. We employed a stratified cluster random sampling method, initially selecting three cities within Jiangxi Province: Pingxiang, Yingtan, and Ganzhou. In each city, kindergartens were randomly chosen from urban and rural regions. Three kindergartens per city were selected, with each enrolling 120 children. Recruitment involved selecting 40 children from each large, medium, and small class in every kindergarten. The study, conducted between March and July 2021, involved 1080 preschoolers. Inclusion criteria were stringent, focusing on age-appropriate, healthy children without physical limitations or severe medical conditions. Exclusions were made in accordance with these criteria.

### Questionnaire survey

Demographic data—including gender, age, and residential status (urban/rural)—were collected from the preschoolers through structured questionnaires [28]. SP patterns were assessed using the "Children's Sleep Habits Questionnaire" (CSHQ), a validated instrument developed by American pediatric SP specialist Owens, to quantify actual SP duration, encompassing nap times. The CSHQ is a widely accepted tool for both diagnosing and researching SP disorders in young children [29, 30]. All questionnaires were completed by the young children's guardians.

### Measurement of physical activity duration

Physical activity duration in preschoolers was meticulously captured using a triaxial accelerometer (ActiGraph wGT3X-BT, USA), affixed to the right iliac crest. The research protocol mandated seven consecutive days of accelerometer wear, inclusive of five weekdays and two weekend days, barring water activities like bathing and swimming. Data extraction and comprehensive analysis were performed on the eighth day using ActiLife v6.13.4 software.

### Classification of physical activity categories in preschoolers

In preschool children, physical activity behaviors are typically classified into four main categories: SB, LPA, Moderate Physical Activity (MPA), and Vigorous Physical Activity (VPA). Based on this categorization, further calculations can be made to determine the MVPA, which is the sum of MPA and VPA, and the Total Physical Activity (TPA), which encompasses LPA, MPA, and VPA. It is recommended for researchers in this field to utilize the Buttle cut point method along with a 15-second sampling interval. This interval has been proven effective for capturing the range of activity levels in preschool-aged children [31].

The classification of physical activity intensity is based on the Buttle (2013) Algorithm. According to this algorithm, SB is characterized by activity levels equal to or less than 239 counts per minute. LPA is defined as activity in the range of 240 to 2119 counts per minute. MPA is determined as activity ranging from 2120 to 4449 counts per minute, while VPA comprises activities that achieve 4450 counts per minute or more. These cut-points have been validated as providing reliable classification accuracy for children aged 3 to 6 years. SP duration was ascertained from the CSHQ, and the remaining time was adjusted based on the proportions of SB, LPA, and MVPA [19].

### Anthropometrics

Measurements were conducted within the kindergarten premises using the "Jianmin" brand, the official equipment for national physical fitness monitoring, following the "National Physical Fitness Measurement Standard Manual" for preschoolers by the General Administration of Sport of China in 2020. Height and weight data were collected, with precision up to one decimal place. Overweight and obesity criteria were based on the "Chinese Children's Obesity Diagnosis, Evaluation, and Management Expert Consensus" [32].

BFP was determined using the BIA method, with the GMCS-3 tester. Participants were guided to stand barefoot on the instrument, ensuring even contact with the electrode pads. After stabilization, they held the electrode handles, extending their arms slightly apart, until the test concluded.

### Data analysis strategy

***Statistical software and basic tests***

IBM SPSS Statistics 25.0 was used for statistical analysis to examine the differences in BFP among preschool children based on gender, age, and urban/rural settings. The χ^2^ test was used to analyze the relationship between non-overweight/non-obese, overweight, and obese preschool children and gender, age, and urban/rural settings. The independent sample t-test was used to analyze the BFP differences among children of different ages. The significance level was set at (*P*<0.05).

#### Compositional data and regression modeling

The geometric mean and variance-covariance matrix described the compositional data's central tendency and dispersion. The log-ratio transformation addressed constraints and correlation issues. Proportions between PA, SB, and SP time were analyzed, and their variances were computed. A linear regression model studied the relationship between the log-ratio transformed activity behavior combination and BFP.

Preschoolers were categorized based on weight to explore physical activity's role. The Compositions and robCompositions packages in RStudio were used. BFP was converted to a log-ratio form to obtain zBFP [33], followed by a compositional ISM analysis.

***Weight-based categorization and ISM analysis***

Initially, a regression model was formulated using activity behavior combinations as the independent variable and Body Fat Percentage (BFP) as the dependent variable, quantified by the R^2^ coefficient. Individual linear regression models were then developed for each potential covariate related to BFP. Significant covariates were incorporated into a refined model, which used equidistant log-ratio transformed activity behaviors and associated pivot coordinates as independent variables. The first pivot coordinate indicated the relative impact of individual activity behaviors on BFP. Consistency was observed in the *β* coefficients and R^2^ values across all models.

***Time reallocation and zero-value handling***

The ISM framework assessed the BFP impact of reallocating 15 minutes among MVPA, LPA, SB, and SP, using 10 minutes per day as a substitution unit [34–36]. Compositional data needs to undergo a log-ratio transformation for analysis, which requires strictly non-zero observations. Since all types of physical activity time for children are involved, zero values are generally not present in the dataset. If zero values appear, they are replaced using the EM algorithm based on ilr coordinates [37].

## Result

### Basic characteristics of participants' body fat percentage and obesity

During the testing phase, data exclusions occurred due to participant withdrawals, missing entries, and invalid questionnaires, leaving 881 valid samples. The survey (Table 1) revealed an average BFP of 18.7% for toddlers. A decline in BFP was noted with age. A notable difference in BFP was observed between urban (18.2%) and rural (17.8%) toddlers, with the former being significantly higher (*P*<0.05). Gender-wise, girls consistently exhibited a higher BFP than boys in the same age bracket (Table 2). An independent sample t-test highlighted significant BFP variations across age groups (F=6.657, *P*<0.05). For ages 3-5, urban toddlers had a higher BFP than their rural counterparts, a trend absent in 6-year-olds. When juxtaposed with national averages, the BFP for all age groups, barring 3-year-olds, was significantly below the standard (*P*<0.05).

By analyzing the overweight/obesity detection rate of preschool children in various age groups (Table 2), it was found that there is no significant difference between different ages (*P*=0.199), but there are significant differences in terms of gender and urban-rural areas *P*<0.01. This indicates that gender and urban-rural factors may affect the weight of preschool children. The overweight/obesity detection rates of preschool children are 10.6% and 7.6%, respectively. Both of these rates are higher than the average overweight and obesity prevalence rates of Chinese preschool children. The urban-rural difference is closely related to the occurrence of obesity in toddlers (*P*<0.05).

**Table 1:** Basic characteristics of preschool children's body fat percentage [%]

Age	Urban/Rural		Gender		Nationwide		Total
	Urban	Rural	Boys	Girls	Boys	Girls	
3 years old	20.9	19.8	18.5^*^	22.3	19.2	23.0	20.4
4 years old	19.1^*^	18.0	17.1^*#^	20.3^#^	18.7^#^	22.0	18.7
5 years old	19.4^*^	17.3	17.5^*#^	20.0^#^	19.7^#^	22.1	18.6
6 years old	18.2	17.1	16.8^*#^	18.8^#^	19.2	21.2	17.7

^*^ indicates independent sample t-test, compared with girls of the same age, *P* < 0.05; # indicates single sample t-test, compared with the same age and gender nationwide, *P* < 0.05

**Table 2:** Univariate analysis of overweight and obesity in preschool children[n(%)]

Feature	Number of Participants (%)	Non-overweight/Non-obese (%)	Overweight (%)	Obese (%)	χ^2^	*P*
Age					8.6	0.199
3years old	116(13.2)	101(87.1)	11(9.5)	5(4.3)		
4years old	284(32.2)	236(83.1)	33(11.6)	15(5.3)		
5years old	271(30.8)	218(80.4)	28(10.3)	25(9.2)		
6years old	210(23.8)	167(79.5)	21(10.0)	22(11.0)		
Gender					9.7	0.008
Boy	465(52.8)	365(78.5)	53(11.4)	47(9.8)		
Girl	416(47.2)	356(85.6)	40(9.6)	20(4.8)		
Urban/Rural					10.3	0.005
Urban	540(61.3)	429(79.4)	58(10.7)	53(9.8)		
Rural	341(38.7)	292(85.6)	35(10.3)	14(4.1)		

### 24-Hour activity behavior

Table 3 presents both arithmetic mean and compositional geometric mean results. Discrepancies exist between the two in representing central tendencies. The arithmetic mean slightly inflates MVPA and LPA durations while downplaying sedentary and SP durations. Compositional geometric averages for SB, LPA, MVPA, and SP stand at 501.6 (34.8%), 213.3 (14.8%), 54.9 (3.8%), and 670.1 (46.5%) respectively, whereas arithmetic averages are 501.0 (34.8%), 214.8 (14.9%), 57.2 (4.0%), and 667.1 (46.3%).

**Table 3:** Compositional geometric mean and arithmetic mean of physical activity, sedentary behavior, and sleep time within 24 h

Compositional Geometric Mean				Arithmetic Mean			
SB/min	LPA/min	MVPA/min	SP/min	SB/min	LPA/min	MVPA/min	SP/min
501.6	213.3	54.9	670.1	501.0	214.8	57.2	667.1
34.8%	14.8%	3.8%	46.5%	34.8%	14.9%	4.0%	46.3%

### Relationship between activity behavior and BFP

Relationship between 24h activity behavior and body fat percentage of participants

Variance matrix results (Table 4) show the smallest variance (0.29) between SB and SP, indicating a robust positive correlation. In contrast, MVPA and SP exhibit the largest variance (2.50), suggesting a mild negative correlation. A significant variance (2.21) between SB and MVPA points to a weaker negative correlation, while a smaller variance (1.36) between LPA and MVPA indicates a stronger positive correlation. As MVPA rises, SP tends to decrease proportionally, and vice versa.

**Fig. 1:**
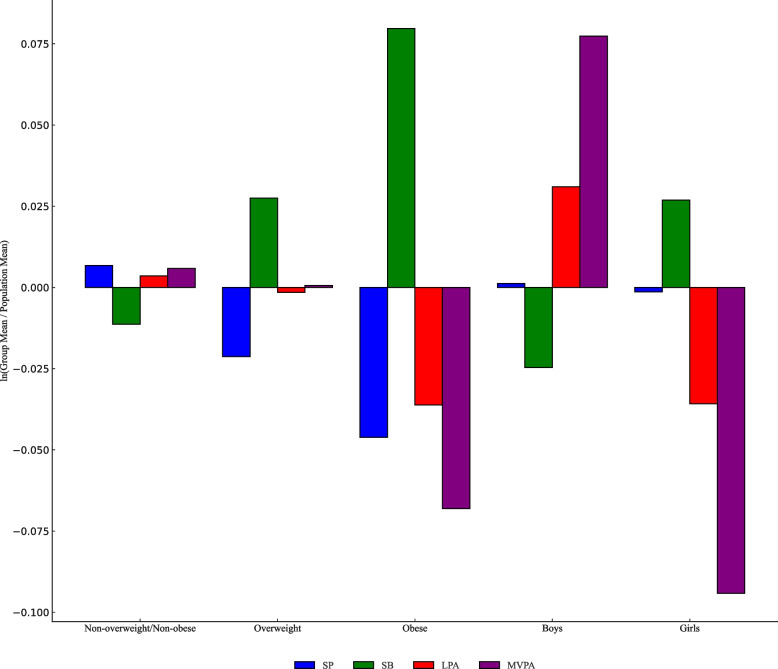
Geometric mean bar graph of the proportion of 24-h activity behaviors n young children

To understand the link between toddler activity behavior and body type, we compared the log-ratio of 24-hour behavior (SB, LPA, MVPA, and SP) across body types against the sample mean using compositional geometric mean bar charts. Results indicate that non-overweight/non-obese toddlers have above-average SP and MVPA levels and below-average SB, with LPA showing no significant deviation. Overweight toddlers exhibit higher SB and lower SP, with LPA and MVPA aligning with the average. Obese toddlers have the highest SB and the lowest SP, LPA, and MVPA. Moreover, boys surpass girls in LPA and MVPA, while girls lead in SB (Figure 1).

**Table 4:** Compositional variation matrix of proportions of time spent in physical activity, sedentary behavior and sleep

	SB	LPA	MVPA	SP
SB	0	0.86	2.21	0.29
LPA	0.86	0	1.36	1.14
MVPA	2.21	1.36	0	2.50
SP	0.29	1.14	2.50	0

### Compositional data regression analysis

Utilizing a Compositional regression model, we examined the association between 24-hour activity duration and zBFP, accounting for potential confounders such as gender, age, and urban-rural disparities (refer to Table 5). The 24-hour activity behavior accounted for 22.8% of the zBFP variability across the sample (model *P*<0.01, R^2^=0.228). Notably, MVPA and LPA proportions exhibited a significant inverse relationship with zBFP, whereas SB proportion displayed a direct relationship. The SP proportion's relationship with zBFP was not statistically significant.

A subgroup analysis highlighted variations in the 24-hour activity behavior's impact on zBFP across different toddler body types. For overweight and obese groups, the positive association between SB proportion and zBFP was more pronounced, particularly in the obese group (R^2^=0.519). In contrast, for the non-overweight/non-obese group, the model accounted for 27.1% of the variability, with SB proportion having a significant direct relationship with zBFP. The MVPA proportion's relationship with zBFP was not significant in both non-overweight/non-obese and obese groups.

Gender differences were evident in the association between 24-hour activity behavior and zBFP. For boys, MVPA proportion had a significant inverse relationship with zBFP, while SB proportion showed a direct relationship. For girls, only the SB proportion was directly related to zBFP, suggesting that MVPA influences boys' zBFP more, whereas LPA has a greater impact on girls.

**Table 5:** Liner regression analysis with compositional data for zBFP of the time spent in physical activity, sedentary behavior and sleep

Category	Model	β	*P*	SE	Model *P*-value	Model R^2^
Overall	ilr_1_-MVPA/(SP*SB*LPA)	-0.114	< 0.05^*^	0.049	< 0.01^**^	0.228
	ilr_1_-LPA/(MVPA*SP*SB)	-0.382	< 0.01^**^	0.087		
	ilr_1_-SB/(LPA*MVPA*SP)	0.681	< 0.01^**^	0.083		
	ilr_1_-SP/(SB*MVPA*LPA)	0.158	0.123	0.101		
Non-overweight/Non-obese	ilr1-MVPA/(SP*SB*LPA)	-0.064	0.173	0.047	< 0.01^**^	0.271
	ilr1-LPA/(MVPA*SP*SB)	-0.177	< 0.05^*^	0.084		
	ilr1-SB/(LPA*MVPA*SP)	0.322	< 0.01^**^	0.079		
	ilr1-SP/(SB*MVPA*LPA)	0.082	0.378	0.093		
Overweight	ilr1-MVPA/(SP*SB*LPA)	-0.239	< 0.01*	0.071	< 0.01^**^	0.443
	ilr1-LPA/(MVPA*SP*SB)	-0.181	0.208	0.142		
	ilr1-SB/(LPA*MVPA*SP)	0.612	< 0.01^*^	0.149		
	ilr1-SP/(SB*MVPA*LPA)	0.446	< 0.05^*^	0.191		
Obese	ilr1-MVPA/(SP*SB*LPA)	-0.061	0.611	0.120	< 0.01^**^	0.519
	ilr1-LPA/(MVPA*SP*SB)	-0.768	< 0.01^**^	0.185		
	ilr1-SB/(LPA*MVPA*SP)	1.194	< 0.01^**^	0.170		
	ilr1-SP/(SB*MVPA*LPA)	0.408	0.100	0.244		
Boys	ilr1-MVPA/(SP*SB*LPA)	-0.155	< 0.05^*^	0.077	< 0.01^**^	0.143
	ilr1-LPA/(MVPA*SP*SB)	-0.394	< 0.01^**^	0.136		
	ilr1-SB/(LPA*MVPA*SP)	0.734	< 0.01^**^	0.122		
	ilr1-SP/(SB*MVPA*LPA)	0.238	0.135	0.159		
Girls	ilr1-MVPA/(SP*SB*LPA)	-0.057	0.33	0.059	< 0.01^**^	0.161
	ilr1-LPA/(MVPA*SP*SB)	-0.398	< 0.01^**^	0.109		
	ilr1-SB/(LPA*MVPA*SP)	0.636	< 0.01^**^	0.109		
	ilr1-SP/(SB*MVPA*LPA)	0.080	0.653	0.122		

The β value refers to the strength of the association between the change in a given behavior relative to other behaviors and zBFP. For example, ilr1-MVPA/(SP*SB*LPA) refers to the strength of the association between zBFP and the time spent in moderate-to-vigorous physical activity (MVPA) as the first coordinate, relative to the time spent in sleep (SP), sedentary behavior (SB), and light physical activity (LPA). SE refers to the standard deviation. All models have been adjusted for factors such as toddler gender, age, and urban or rural areas

### Reallocating of 24-Hour activity duration and its impact on zbfp prediction

The association observed between redistributing 15 minutes among 24h activities and zBFP, considering the influences of gender, age, and region, is detailed in Table 6 for toddlers. It was found that reallocating time from SB to LPA or MVPA is significantly associated with a decrease in zBFP (ΔzBFP =-0.030 and -0.034, *P*<0.05). Conversely, reallocating time from SP to SB shows a significant association with an increase in zBFP (ΔzBFP =0.026, *P*<0.05). Toddlers of different body types respond differently to activity intensity. In the non-overweight/non-obese group, time shifted from SB to LPA or MVPA is significantly associated with a decrease in zBFP (ΔzBFP=-0.014 and -0.018, *P*<0.05), while time shifted from SP to SB is significantly associated with an increase in zBFP (ΔzBFP=0.012, *P*<0.05). In the overweight group, time shifted from SB to LPA or MVPA is significantly associated with a decrease in zBFP (ΔzBFP=-0.018 and -0.057, *P*<0.05), and the effect is greater than in the non-overweight/non-obese group. In the obese group, only the shift from SB to LPA is significantly associated with a decrease in zBFP (ΔzBFP =-0.056, *P*<0.05). It can be seen that a notable association is observed between MVPA and zBFP in overweight toddlers while LPA has a greater effect in obese toddlers.

In terms of gender differences, for boys, time shifted from SB to SP, LPA, or MVPA is significantly associated with a decrease in zBFP (ΔzBFP =-0.26, -0.031, and -0.041, *P*<0.05). For girls, time shifted from SB to LPA and SP is significantly associated with a decrease in zBFP (ΔzBFP =-0.030 and -0.026, *P*<0.05). From a gender perspective, boys' zBFP may be more influenced by changes in MVPA time, while girls are more significantly affected by shifts in LPA and SP time.

**Table 6:** The Effect of 15 min Reallocation between MVPA, LPA, SB, SP on zBFP

					ΔzBFP (95%CI)
Model		SP↓	SB↓	LPA↓	MVPA↓
Overall	SP↑		-0.026(-0.032,-0.020)^*^	0.005(-0.005,0.014)	0.013(-0.012,0.039)
	SB↑	0.026(0.020,0.031)^*^		0.030(0.20,0.041)^*^	0.039(0.014,0.063)^*^
	LPA↑	-0.004(-0.013,0.005)	-0.030(-0.040,-0.020)^*^		0.009(-0.021,0.040)
	MVPA↑	-0.008(-0.028,0.012)	-0.034(-0.052,-0.016)^*^	-0.003(-0.028,0.022)	
Non-overweight/Non-obese	SP↑		-0.012(-0.018,-0.006)^*^	0.002(-0.007,-0.011)	0.009(-0.016,0.034)
	SB↑	0.012(0.006,0.018)^*^		0.014(0.035,0.024)^*^	0.021(-0.003,0.045)
	LPA↑	-0.016(-0.010,0.067)	-0.014(-0.023,-0.004)^*^		-0.008(-0.022,0.037)
	MVPA↑	-0.006(-0.025,0.013)	-0.018(-0.036,-0.001)^*^	-0.004(-0.028,0.020)	
Overweight	SP↑		-0.011(-0.020,-0.002)^*^	0.007(-0.011,0.025)	0.061(0.024,0.098)^*^
	SB↑	0.011(0.002,0.020)^*^		0.018(0.000,0.036)^*^	0.072(0.035,0.109)^*^
	LPA↑	-0.007(-0.024,0.010)	-0.018(-0.035,-0.001)^*^		0.054(0.009,0.100)^*^
	MVPA↑	-0.046(-0.074,-0.018)^*^	-0.057(-0.085,-0.029)^*^	-0.039(-0.077,-0.001)^*^	
Obese	SP↑		-0.037(-0.052,-0.022)^*^	0.021(-0.002,0.045)	-0.003(-0.075,0.068)
	SB↑	0.037(0.022,0.052)^*^		0.058(0.036,0.080)^*^	0.033(-0.035,0.101)
	LPA↑	-0.019(-0.041,0.003)	-0.056(-0.077,-0.035)^*^		-0.023(-0.103,0.058)
	MVPA↑	0.006(-0.049,0.060)	-0.032(-0.082,0.019)	0.027(-0.038,0.092)	
boys	SP↑		-0.026(-0.035,-0.017)^*^	0.005(-0.009,0.020)	0.021(-0.016,0.059)
	SB↑	0.026(0.017,0.034)^*^		0.031(0.015,0.046)^*^	0.047(0.012,0.082)^*^
	LPA↑	-0.004(-0.018,0.009)	-0.031(-0.046,-0.016)^*^		0.017(-0.028,0.062)
	MVPA↑	-0.015(-0.044,0.015)	-0.041(-0.068,-0.014)^*^	-0.009(-0.047,0.028)	
Girls	SP↑		-0.026(-0.034,-0.018)^*^	0.005(-0.007,0.017)	-0.001(-0.037,0.036)
	SB↑	0.026(0.018,0.033)^*^		0.031(0.017,0.044)^*^	0.025(-0.010,0.059)
	LPA↑	-0.004(-0.015,0.007)	-0.030(-0.043,-0.017)^*^		-0.005(-0.047,0.037)
	MVPA↑	0.003(-0.024,0.030)	-0.023(-0.049,0.002)	0.008(-0.026,0.042)	

↑ indicates an increase of 15 min in the activity duration

↓ indicates a decrease of 15 min. Adjustments were made for covariates such as age, gender, and urban–rural differences in the model

^*^ indicates *P* < 0.05. The zBFP regression coefficient indicates that when controlling for covariates, the change in zBFP due to equal time substitution of activity components is β*100%

### Dose–response relationship of 24-h activity behavior substitution with zBFP

**Fig. 2:**
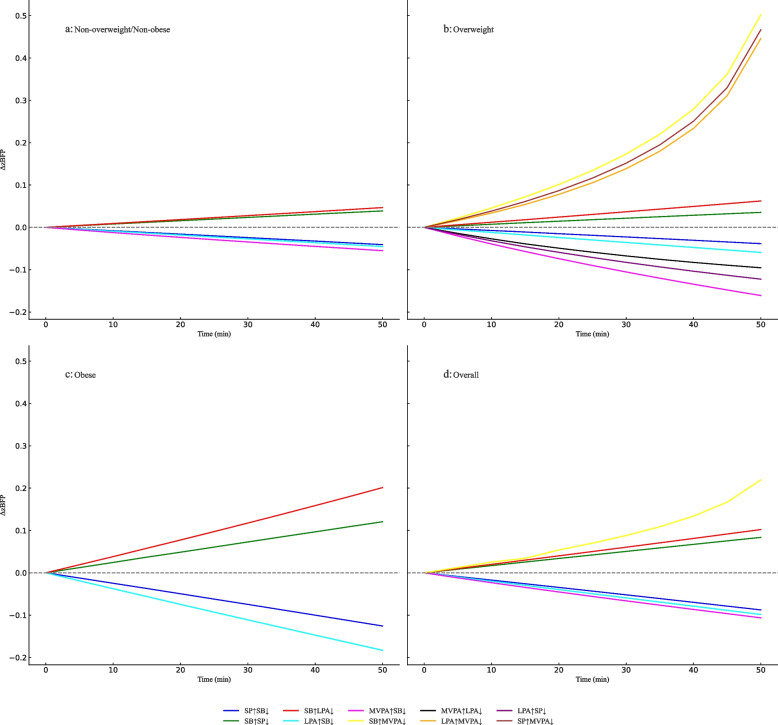
The impact of 24-h isotemporal substitution of physical activity behaviors on zBFP in preschoolers with different body types

**Fig. 3:**
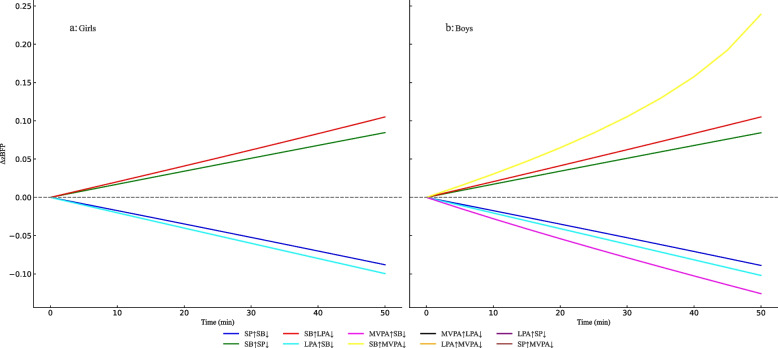
Differences in the impact of 24 h activity behavior substitution on zBFP between genders

To further explore the dose-response between different activity behaviors substituting for each other and BFR, significant activity elements affecting zBFR were selected. With a substitution increment of 5 minutes, extending the duration to 50 minutes, the ISM results show a clear asymmetry in the dose-response relationship between MVPA and LPA, SB, and SP. Specifically, when MVPA replaces other activities, zBFR shows a slow decreasing trend; conversely, when other behaviors replace MVPA, zBFR rises rapidly. Notably, regardless of gender or body type, the dose-response relationship between SP and SB and between SB and LPA is essentially symmetrical (Figures 2-3).

In the analysis of toddlers of different body types (Figure 2), the zBFR dose-response in the non-overweight/non-obese group is relatively stable. In contrast, the overweight group has a greater effect on activity behavior substitution, manifested as a significant increase in the rate of change of zBFR. Especially when MVPA time decreases by 30 minutes, zBFR rises rapidly. This suggests that overweight toddlers should pay more attention to ensuring their daily MVPA time (>30min/d has a greater effect on reducing zBFR). In the obese group, the rate of change of zBFR is higher when SB and LPA substitute for each other, and the decrease in zBFR when SB and SP replace other behaviors is also higher than in other groups.

In terms of gender (Figure 3), boys have a faster increase in zBFR when other behaviors replace MVPA, and zBFR gradually decreases when MVPA replaces other behaviors, with the greatest impact being from SB. For girls, the substitution effect between SB and LPA is particularly significant. Moreover, the dose-response relationship between SP and SB for zBFR is similar to that of boys.

## Discussion

This study utilized the compositional isotemporal substitution model to examine the association between 24-hour activity behavior and body fat percentage (BFP) in preschool children. The findings indicate that 24-hour activity behavior is associated with 22.8% of the variation in BFP, and the explanatory power was higher in overweight and obese children. By comparing the 24-hour activity behavior of overweight and obese children with the overall sample, it was found that the sedentary time of overweight and obese children was higher than the average level, and MVPA was lower than that of non-overweight/non-obese children [38–40]. This is consistent with similar studies by other researchers.

Previous research has shown a correlation between high levels of physical activity and lower rates of overweight and obesity in children [41]. This study, however, observes changes in zBFP among overweight children in relation to their 24-hour activity patterns, particularly noting variations associated with engaging in MVPA for more than 30 minutes. Specifically, as MVPA time increased in overweight children, the rate of decrease in BFP was faster than with LPA. However, when MVPA time was reduced and exceeded 30 minutes, BFP rapidly increased. Compared to overweight children, obese children's BFP was more influenced by LPA and sleep time (SP), with a significant increase in BFP as SB replaced these activities. Non-overweight/non-obese children's BFP was less influenced by physical activity (PA) [24]. This trend is broadly similar to the one observed by Fairclough SJ et al. [42], who analyzed compositional data across different weight status categories for changes in cardiorespiratory fitness (CRF). The phenomenon might be attributed to the significant increase in energy expenditure with MVPA, which for overweight children means more effective fat burning and reduction in BFP.

Research documents associations between the insulin/insulin-like growth factor signaling axis and chronic low-grade inflammation with the development of obesity in children, alongside observations of specific adipocyte factors like leptin, adiponectin, and resistin, which are closely associated with energy metabolism and inflammation regulation. PA can reduce the infiltration and activation of inflammatory cells in adipose tissue, lower the levels of circulating inflammatory factors, enhance the activity of antioxidant enzymes, and decrease the formation of oxidative damage products. This effectively improves insulin sensitivity and lipid levels, offering multiple pathways to regulate inflammation and oxidative stress responses associated with childhood obesity [43]. Furthermore, studies have observed that MVPA is associated with reductions in obesity and its related complications through multiple pathways, including associations with increased energy expenditure, improved metabolic health, regulated adipocyte factors, reduced chronic inflammation, enhanced cardiopulmonary function, and positive psychological and behavioral changes [44].

For obese children, due to the difficulty in participating in MVPA, improvements in LPA (such as walking, jogging) and sleep quality may present more practical ways to manage weight and health. However, this contrasts with studies on children of different ages, where Dumuid D et al [25]. observed a notable association between MVPA in the obese group, likely due to their generally lower levels of MVPA in daily life. The significant influence of obesity on physical capability and activity levels means that these children may see more marked improvements in BFP with increased MVPA. A higher intensity or longer duration of MVPA may be required to produce similar effects [38].

The study found that male children exhibited higher levels of MVPA and LPA than females and were more affected by changes in MVPA. Conversely, female children’s BFP was more influenced by LPA and SP [13, 45]. In different gender groups, male children showed a significant increase in BFP after 30 minutes when SB replaced MVPA. This aligns with a study on 9-11-year-old children using a similar compositional data isotemporal substitution analysis, but in contrast to 3-6-year-old children, the increase in BFP occurred later, around 45 minutes, indicating that younger children are more sensitive to reductions in physical activity. Additionally, in this study, female children did not exhibit a rapid increase in BFP when other activities replaced MVPA, contrasting with findings by Dumuid D et al., where a sharp increase was observed after 50 minutes in 9-11-year-old children. The average MVPA time in female toddlers did not reach this duration, preventing exploration of isotemporal substitution effects beyond 50 minutes. Thus, significant behavioral activity time differences are evident between children of different age groups, and similar conclusions from research cannot be directly extrapolated [23].

Although many studies have established a connection between sleep duration (SP) and childhood obesity [27, 46], some suggest that the quality and consistency of SP are more critical determinants of obesity [47]. This study found this association primarily in overweight children, implying that other factors might overshadow the SP-obesity relationship in non-overweight/non-obese children. Evidence suggests that severely obese children often have later SP times, correlating with increased screen time and decreased PA, both contributing factors to obesity [48].

In conclusion, this research highlights the inverse relationship of MVPA and LPA with BFP in preschoolers, while SB correlates directly. Associations suggest that higher levels of MVPA and LPA, coupled with reduced SB, correlate with lower BFP in preschool children. To address the growing obesity epidemic, broader initiatives, such as mandatory dietary standards in educational institutions and improved community recreational facilities, are advocated [49].

This study has inherent limitations. Firstly, due to its cross-sectional design, it can only reveal correlations and not establish causality. Secondly, while accelerometers were used to measure the physical activity of toddlers, these devices cannot differentiate activity types or capture upper limb movements. Additionally, the method used for measuring body fat, bioelectrical impedance analysis, is convenient but can be influenced by factors such as hydration and diet and does not differentiate between types of fat. The study also did not consider food intake, leaving the role of diet in observed associations undetermined. Furthermore, the sample was limited to 3-to-6-year-old children in Jiangxi Province. Children in different regions may have varying lifestyles, dietary patterns, and socio-cultural backgrounds, which can impact physical activity behavior and BFP. Therefore, the findings of this study may not be applicable to children from other regions or with different socio-economic backgrounds. Lastly, the complex interplay between obesity and psychological issues (such as low self-esteem and depression) complicates the understanding of the causes and effects of childhood obesity.

## Conclusions

Our findings underscore a strong association between 24-hour activity behavior and BFP in preschool children, particularly pronounced in those who are overweight or obese. Using compositional ISM, we observed a distinct asymmetry in the dose-response relationship when substituting MVPA with LPA, SB, or SP. MVPA emerged as the most significant modulator of BFP in overweight children, with a 30-minute daily duration yielding the most substantial reduction. Interestingly, the impact of MVPA on BFP showed gender-specific patterns: males were predominantly influenced by MVPA, whereas females showed stronger correlations with LPA and SP. 

## Data Availability

The datasets generated and/or analyzed during the current study are available from the corresponding author on reasonable request.

## Abbreviations

SP: Sleep
SB: Sedentary Behavior
PA: Physical Activity
LPA: Light Physical Activity
MPA: Moderate Physical Activity
MVPA: Moderate to Vigorous Physical Activity
TPA: Total of Physical Activity
BFR: Body Fat Percentage
ISM: Isotemporal Substitution Model
CoDA: Compositional Data Analysis
CSHQ: Children’s Sleep Habits Questionnaire
CRF: Cardiorespiratory Fitness
